# Effective implantation of autologous chondrocytes in a patient suffering from a painful and invalidating rizoarthrosis: a case report

**DOI:** 10.4076/1757-1626-2-7886

**Published:** 2009-08-18

**Authors:** Francesco Carelli, Stefano Sgherzi, Alessandro Sillani, Cecilia Magris

**Affiliations:** School of Medicine, University of MilanVia Festa del Perdono 7, 20122 MilanoItaly

## Abstract

A 45-year-old patient, caucasian, affected by severe, painful and invalidating rizoarthrosis has been treated by implanting autologous chondrocytes, normally used for degenerative joint diseases of the knee and ankle.

## Case presentation

The patient is a 45-year-old Caucasian woman known to suffer from rizoarthrosis, who has already undergone antidolorific and anti-inflammatory therapy (oral and systemic), as well as physiotherapy and other treatment such as ionophoresis, ultrasound and electrotherapy. She comes to our surgery and complains that the pain in her right hand is becoming more and more severe, with loss of the joint function, which is not only impeding her work, but also restricts her daily activities.

Therefore we had to think about a more effective therapy. She has been finally treated by implanting autologous chondrocytes, normally used for degenerative joint diseases of the knee and ankle.

Because of the young age of the patient we were doubtful about using the classical procedures (trapeziectomy, prosthesis, etc.), preferring to apply a less invasive technique which could guarantee good movement and alleviation of the pain in the case of success, but without compromising the possible need to resort to conventional therapies in the event of failure.

We therefore read up widely on the different therapeutic possibilities for rizoarthrosis and were impressed by the encouraging achievements reported by the literature of a therapy which is still in the experimentation phase and involves the use of autologous chondrocytes. In our opinion this could be the most appropriate for our patient.

We informed her about this new possibility, and it was agreed that we should undertake this new type of treatment.

Our patient was then examined in the outpatients’ department of microsurgery of the hand at the Gaetano Pini Hospital in Milan, and the orthopaedic surgeon confirmed that she would be an ideal candidate to take part in the clinical trial.

She therefore joined the first clinical trial, the goal of which was to assess the application of the technique of autologous chondrocytes transplant in small joints at the Gaetano Pini Hospital in Milan.

The treatment was carried out in March 2008, and it seems to have been successful: for the moment the patient is enjoying good health.

The joint cartilage covers the articular bones in order to protect them from friction and allow them to move.

The implant of autologous chondrocytes (Tissue Engineering) becomes a valid alternative to the classical treatments employed in cases of joint cartilage damage in patients destined for invalidity or for prosthetic replacement of the damaged bones [1-3].

Because of its particular architecture (few cells situated far apart, surrounded by a dense matrix, and absence of vessels and nerves), the hyaline cartilage is not able to regenerate itself.

Following damage, it can sometimes start a reparative process, which leads to the formation of fibrous cartilage (collagen type I) with characteristics of resistance and deformability very different from those presented by the original hyaline cartilage (collagen type II). Consequently, it will originate a degenerative arthrosic pathology accompanied by the onset of chronic pain and functional limitation [4,5].

The symptoms are mostly movement difficulties due to pain, swelling, hemartrosis and at times joint block.

Surgical treatment is recommended above all in those cases in which pain is the predominant symptom and persists in spite of the use of antidolorific therapy.

The Mosaic Graft and the Microfracture are surgical techniques which present the disadvantage of originating fibrous cartilage, which is not as resistant and functional as the hyaline cartilage, and can be performed in arthroscopy only in the case of small lesions (<3 cm²).

The implant of autologous chondrocytes has shown that it can induce the regeneration of hyaline cartilage, even if the joint lesions are deep [6,7]. In fact its trophism does not depend on blood flow, but is guaranteed directly by the synovial liquid. Nor is innervation necessary.

A small fragment of healthy cartilage tissue is taken by means of arthroscopy from an area not subjected to load and is sent to a laboratory for replication of the cartilage cells. There it undergoes enzymatic digestion, followed by isolation and cell culture. After nearly a month of growing, the chondrocytes can be transplanted [8-10].

The cells, adhering to a collagen membrane, are implanted into the patient using a fibrin adhesive, absorbable sutures or resorbable pins.

During the following weeks and months the membrane will dissolve and the chondrocytes produce the matrix for the cartilage’s regeneration.

As with other surgical procedures, the capital moment is the post-surgery rehabilitation of the patient. The stay in hospital will be around 5-6 days. The day after surgery the patient starts the programme of functional re-education with passive mobility of the joint. Active exercises start after 25-30 days, with progressive loading beginning after 30 days and completed after 3 months with proprioceptive and functional re-education exercises.

Low levels of exercise can be started after 5 months and can then be increased after 1 year.

Although the new cartilage develops after a few weeks, it is better to avoid prolonged effort for a period of at least 4-6 months in order to allow perfect maturation of the new tissue.

The implant technique of autologous chondrocytes was developed in Sweden by Brittberg and colleagues and has already been used in many European and non-European countries for about ten years. It achieves a high percentage of recovery in joints such as the knee or ankle.

Recently this technique has been employed in the treatment of rizoarthrosis and articular damage to the hand and wrist, with more benefits in comparison to classical techniques.

Even if arthroplasty of the trapezium-metacarpal articulation with trapeziectomy achieves good results in terms of pain relief and mobility, it involves a loss of strength due to trapezium removal, and after some years this could lead to instability of the index finger.

Arthrodesis resolves the pain and maintains the strength, but induces a limitation of mobility and can involve intercarpal arthrosic degeneration.

The use of a prosthesis has shown good results, but these are not always long-lasting.

The risks that could occur in the implantation of autologous chondrocytes are the same as with all surgical interventions (septic, thromboembolic and anestesiologic complications etc.). There is a non-predictable risk, including that due to cartilage manipulation, allergic reactions to the buffer solution employed in the conservation of the product, the risk of loss of the initial benefits after a time, or the risk that the intervention turns out to be unsuccessful.

In the event of failure it is still possible to resort to the classical techniques, since the implantation of autologous chondrocytes does not rule out the possibility of subsequent traditional treatment.
